# Sex-specific disease outcomes of HIV-positive and HIV-negative drug users admitted to an opioid substitution therapy program in Spain: a cohort study

**DOI:** 10.1186/1471-2334-14-504

**Published:** 2014-09-17

**Authors:** Roberto Muga, Inmaculada Rivas, Eva Faure, Daniel Fuster, Paola Zuluaga, Manuela Rubio, Trinidad Muñoz, Marta Torrens, Jordi Tor, Arantza Sanvisens

**Affiliations:** Department of Internal Medicine, Hospital Universitari Germans Trias i Pujol, Universitat Autònoma de Barcelona, 08916 Badalona, Spain; Municipal Centre for Substance Abuse Treatment (Centro Delta), Badalona, Spain; Section of General Internal Medicine, Boston Medical Center, Boston University School of Medicine, Boston, MA USA; Institute of Neuropsychiatry & Addictions, Parc de Salut Mar, Universitat Autònoma Barcelona, Barcelona, Spain

**Keywords:** Methadone, Opioid substitution treatment, Mortality, HIV

## Abstract

**Background:**

Opioid substitution therapy has improved the survival of heroin users with and without HIV infection. We aimed to analyze sex differences in mortality rates and predictors of death among those admitted to a methadone treatment program (MTP).

**Methods:**

Longitudinal study of patients enrolled in a MTP from 1992 to 2010. Socio-demographic and drug use characteristics, and markers of viral infections were assessed at entry. Vital status was ascertained by clinical charts and the mortality register. Four calendar periods were defined according to the introduction of preventive and treatment interventions in Spain. Predictors of death were analyzed by Cox regression models.

**Results:**

1,678 patients (82.8% men) were included; age at first heroin use was 18.6 years (IQR: 16–23 years), and age at first entry into a MTP was 30.7 years (IQR: 26–36 years). A total of 441 (26.3%) deaths occurred during 15,124 person-years (p-y) of follow-up (median: 9.2 years, IQR: 4–13 years). HIV infection was the main predictor of death in men (HR = 3.5, 95% CI: 2.1-5.7) and women (HR = 3.2, 95% CI: 1.2-8.7 ) and main cause of death was HIV/AIDS. Overall mortality rate was 2.9 per 100 p-y (95% CI: 2.7-3.2 per 100 p-y) and death rates decreased over time: 7.4 per 100 p-y (95% CI: 6.3-8.8 per 100 p-y) for the 1992–1996 period to 1.9 per 100 p-y (95% CI: 1.6-2.4 per 100 p-y) for the 2007–2010 period. In women, a slightly increase in mortality was observed in recent periods specifically among HIV-positive women (3.7 per 100 p-y in period 2002–2006 and 4.5 per 100 p-y in 2007–2010).

**Conclusions:**

Significant reductions in mortality of patients in MTP are observed after nineteen years of observation. However, HIV infection shows a great impact on survival, particularly among HIV-infected women.

**Electronic supplementary material:**

The online version of this article (doi:10.1186/1471-2334-14-504) contains supplementary material, which is available to authorized users.

## Background

It has been estimated that 1.35 million people are heroin users in European countries [1]. Heroin dependence is a chronic disease characterized by compulsive drug-taking behavior despite serious negative consequences and, specifically, injection drug users (IDUs) have high prevalence of severe co-morbidities including blood-borne infectious diseases, drug overdose, and psychiatric disorders [2–4].

The efficacy of detoxification as the main treatment intervention is limited, as compared to other therapeutic options such as opioid substitution therapy (OST) [5]. OST reduces heroin consumption by acting as opioid receptor agonists on the central nervous system. A reduction in heroin use further increases adherence to substitution therapies and facilitates the implementation of harm-reduction interventions aimed to improve quality of life and health [5–9]. Such health and social interventions while in substitution therapy are common for Methadone Treatment Programs (MTP). MTPs have been used since the mid-1960 in the United States (U.S.) [10] and more recently in Western Europe. Over 20 years of scientific evidence has demonstrated the effectiveness of OST on reducing the impact of illegal opiate usage in society [11, 12]. Most importantly, treatment with methadone has been shown to reduce the risk of death as compared to no treatment [13]. Treatment with methadone also reduces the risk of blood-borne infections such as human immunodeficiency virus (HIV); methadone treatment in combination with needle exchange programs and other harm reduction strategies has been shown to reduce the risk of hepatitis C virus (HCV) infection [14–16].

In Western Europe, the experience of OST for the treatment of heroin dependence is heterogeneous. In most countries, including Spain, methadone is the preferred drug for OST, while buprenorphine is more common in others [17]. Approximately 90,000 patients receive methadone treatment in Spain, and this figure has remained constant since the early 1990’s, when OST was focused to reduce the impact of HIV/AIDS and drug overdose [17]. Spanish drug users were heavily impacted by HIV infection to a point that there was a reduction in life expectancy for the general population during the early 1990's [18]. Despite the impact that heroin dependence had in young adults from urban settings, studies examining long-term disease outcomes from MTPs in Spain are scarce [7, 19].

The aim of this study was to analyze the survival of heroin users and predictors of death in a cohort of patients admitted to a MTP. Specifically, we aimed to analyze sex differences in mortality, as well as temporal changes over four calendar periods, which are characterized by the introduction of preventive and treatment interventions in metropolitan Barcelona, Spain. In doing so, our results will inform how MTP is implemented in our area.

## Methods

A longitudinal study of patients admitted to a MTP from January 1992 to December 2010 was performed. This MTP began in January 1992, is the only OST in the metropolitan area that is north of Barcelona, and is located in the CAS-Delta, Municipal Institute for Personal Services, in Badalona. The CAS-Delta is a primary care center for outpatient addiction treatment, and serves an area with 400,000 inhabitants over five municipalities, including Badalona (240,000 inhabitants) and Santa Coloma de Gramenet (120,000 inhabitants). Within the program, methadone is dispensed via a primary care center, a mobile unit since 1993 and five community pharmacies since 1999.

MTP admission criteria were as follows: patients had to be >18 years old, and had to have an opioid dependence according to the Diagnostic and Statistical Manual of Mental Disorders, 4^th^ edition (DSM-IV) criteria. Psychosocial interventions and harm reduction programs, such as needle exchange, condom distribution, and supervised injection facilities, complemented the drug treatment program. Patients were referred to other Primary Care outpatient facilities or hospitals in the area for additional studies, if necessary. Additional MTP details have been published elsewhere [20].

Socio-demographic data (age at admission, education level, employment status, and prior imprisonment), drug use characteristics (age at first consumption, duration and route of administration), blood samples to test for HIV, HBV (HBcAb+), and HCV infections, and psychiatric history were collected at MTP admission. Information was obtained from medical records.

The psychiatric disorders were diagnosed by specialist at the MTP or other centers, and were divided into four categories: 1) mood-related disorders, 2) anxiety disorders, 3) schizophrenia or other psychotic disorders, and 4) personality disorders.

To analyze changes over time, four different time periods were established according to the introduction of preventive and treatment interventions in Barcelona, Spain: 1) 1992–1996: characterized by the development of methadone treatment and needle exchange programs. At the same time, antiretroviral therapy (ART) emerged as a treatment for HIV infection with monotherapy or dual therapy of nucleotide analogues [21], 2) 1997–2001: period that saw the widespread introduction of Highly Active Antiretroviral Therapy (HAART), 3) 2002–2006: period when HAART was simplified and supervised injection facilities for IDUs were created [22–24], and 4) 2007–2010: period that represented the consolidation of harm reduction interventions and the treatment of HIV infection with HAART in once-daily dosing.

The present study is compliant with ethical standards for medical research and good clinical practice principles, in accordance with the World Medical Association’s Declaration of Helsinki. The study was approved by the Ethics Committee of the Hospital Universitari Germans Trias i Pujol and written consent of patients was obtained.

### Follow-up, mortality and cause of death

Mortality and cause of death were ascertained by reviewing clinical charts and crosschecking with the Catalonian mortality register, as of December 31st, 2010.

Cause of death was established in accordance with the International Classification of Diseases, Version 9 (ICD-9) until 1998 [25], and Version 10 (ICD-10) between 1999 and 2010 [26]. Causes of death were later classified into four categories:

1. Non-natural (including drug-related and alcohol-related accidents, suicides, and traumas): ICD-9: 304, 305, and E800 - E999; ICD-10: F10 - F19, X00 - X99, V00 - V99, W00 - W99, and Y00 - Y36.
2. HIV/AIDS: ICD-9: 279.5 and 795.8; ICD-10: B20 - B24 and R75.
3. Liver-related (including viral hepatitis, cirrhosis, decompensated liver disease, and hepatocellular carcinoma): ICD-9: 070, 155, and 570–573; ICD-10: B15 - B19, C22, and K70 - K77.
4. Other medical causes, including non-HIV/AIDS-related infectious diseases, and respiratory and central nervous system disorders (codes not specified in other categories).

### Statistical analysis

Descriptive statistics were expressed as the median (interquartile range [IQR]) for continuous variables and as absolute frequencies and percentages for categorical variables. We used χ-square tests, Fisher F-tests, and Student’s t-test to detect significant differences. Patients’ entry to the cohort was defined by the first admission to MTP; all patients were followed until December 31^st^ 2010 or death.

Cox regression models were used to analyze the predictors of death. Previous to the implementation of statistical models we checked for the proportional hazards assumption of all variables. The covariates used for multivariate analysis were those that were found to be statistically significant in univariate analysis. Stepwise forward selection was used to identify predictors establishing α = 0.05 and α = 0.1 as the inclusion and exclusion criteria, respectively.

Mortality rates were calculated in person-years (p-y) analysis; p-y represents the time that all persons contribute to a longitudinal study. Rates of mortality in p-y analysis are the quotient of the number of deaths observed during the study period in the numerator and the sum of all the individual follow-up times in the denominator. Temporal changes in mortality rates were observed for four periods: 1992–1996, 1997–2001, 2002–2006, and 2007–2010.

All analyses were conducted using two-sided tests and a significance level of 0.05. Descriptive statistics and regression models were generated using SPSS 15.0 (SPSS, Chicago, IL, USA). Mortality rates for the calendar years were calculated using STATA version 8.0 (Stata Corp., College Station, TX, USA).

## Results

The study included 1,678 patients (82.8% men). The median age of first heroin use was 18.6 years (IQR: 16–23 years), median age at MTP admission was 30.7 years (IQR: 26–36 years) and 23.8% of patients were non-IDUs. Forty four percent of patients were employed, 83% had completed primary education, and 48% had prior incarceration.

Psychiatric co-morbidities were analyzed for 1,567 patients; 25% (394) were diagnosed with psychiatric disorders, and 22% (87/394) had more than one disorder. The most frequent diagnoses were related to mood disorders in 45.9% (181/394), personality disorders in 34.2% (135/394), and anxiety in 17.7% (70/394). Twelve percent (48/394) of patients were diagnosed with a psychotic disorder, and 8.4% (33/394) had previously attempted suicide.

The characteristics of patients by sex are described in Table 1. Men were more likely to have prior incarceration and women were more likely to be HIV-infected (59.8% vs. 52.5%, *p* = 0.040) and to have psychiatric co-morbidities (30.3% vs. 24.1%, *p* = 0.031), especially mood disorders.

**Table 1:** **Baseline characteristics of 1,390 men and 288 women admitted to a methadone treatment program in metropolitan Barcelona, Spain, between 1992 and 2010**

	Men	Women	***p***value
	n = 1,390	n = 288	
	n (%)	n (%)	
**Age at admission (median, IQR)**	31 (26–36)	30 (24–35)	0.000
**Period of admission:**			0.972
**1992-1996**	505 (36.3)	103 (35.8)	
**1997-2001**	469 (33.7)	97 (33.7)	
**2002-2006**	250 (18.0)	55 (19.1)	
**2007-2010**	166 (11.9)	33 (11.5)	
***Social and drug use characteristics***			
**Age at first heroin use (median, IQR)**	19 (17–22)	19 (16–24)	0.940
**Injection drug use (n = 1,562)**	992 (76.8)	198 (73.1)	0.184
**Unemployed (n = 1,560)**	703 (54.5)	164 (60.5)	0.072
**Primary school attainment (n = 1,552)**	1,065 (83.1)	223 (82.3)	0.735
**Prior incarceration (n = 1,480)**	610 (49.8)	100 (39.4)	0.003
***Co-morbidity***			
**HIV infection (n = 1,343)**	582 (52.5)	140 (59.8)	0.040
**HCV infection (n = 727)**	443 (73.2)	92 (75.4)	0.617
**HBV infection (HBcAb+) (n = 708)**	369 (62.2)	65 (56.5)	0.250
**Psychiatric disorder (n = 1,567)**	311 (24.1)	83 (30.3)	0.031

### Follow-up and outcomes

Overall, the median follow-up time for the entire cohort was 9.2 years (IQR: 4–13 years), while the total follow-up was 15,124 p-y. At the end of follow-up, 441 (26.3%) patients had died and 14 (3.2%) of deaths occurred within one month of first entry to treatment. The overall mortality rate was 2.9 per 100 p-y (95% Confidence Interval (CI): 2.6-3.2 per 100 p-y), and the main causes of death were HIV/AIDS (40%) and non-natural (26.4%) (Table 2). No sex differences were observed for the median follow-up (*p* = 0.097), cumulative mortality (*p* = 0.403), or mortality rate (*p* = 0.187). However, distribution of the causes of death was significantly different between sex (*p* = 0.022); HIV/AIDS-related mortality was higher in women (53.0% vs. 37.3%) and mortality from non-natural causes was higher in men (29.0% vs. 12.1%).

**Table 2:** **Outcomes of interest at the end of follow-up (December 31th, 2010) in 1,678 opiate users admitted to a methadone treatment program between 1992-2010**

	Men	Women	p value
	n = 1,390	n = 298	
**Total follow-up length (p-y)**	12,356	2,768	
**Median follow-up (IQR), years**	9.1 (3.8-12.8)	9.6 (4.9-13.8)	0.097
**Deaths, n (%)**	371 (26.7)	70 (24.3)	0.403
**Mortality Rate, per 100 p-y (95% CI)**	3.0 (2.7-3.3)	2.5 (2.0-3.2)	0.187
**Causes of death (n = 425)**			0.022
**HIV/AIDS**	134 (37.3)	35 (53.0)	
**Non-natural**	104 (29.0)	8 (12.1)	
**Liver**	38 (10.6)	8 (12.1)	
**Other**	83 (23.1)	15 (22.7)	

### Sex-specific predictors of death

In the univariate analyses, age at admission, antecedent of IDU, HCV, HIV and HBV infections at admission, prior incarceration, and period of admission to MTP were associated with mortality among men. In the multivariate analysis, only HIV infection at admission was an independent predictor of death (Table 3).

**Table 3:** **Cox regression models for predictors of death in 1,390 men and 288 women admitted to a methadone treatment program in metropolitan Barcelona, Spain, between 1992 and 2010**

	Men				Women			
	Univariate		Multivariate^1^		Univariate		Multivariate^2^	
	HR	CI 95%	HR	CI 95%	HR	CI 95%	HR	CI 95%
**Age at admission**	**1.03**	**1.02-1.05**			**1.04**	**1.00-1.08**		
**Period of admission**								
**1992-1996**	**2.33**	**1.18-4.59**			1.24	0.28-5.44		
**1997-2001**	1.31	0.66-2.62			0.90	0.20-4.02		
**2002-2006**	0.96	0.45-2.01			0.86	0.17-4.21		
**2007-2010**	1				1			
***Social and drug use characteristics***								
**Age at first heroin use**	1.01	0.99-1.03			1.02	0.97-1.07		
**Injection drug use**	**2.87**	**1.97-4.18**			**7.38**	**2.32-23.52**		
**Unemployed**	1.18	0.95-1.46			0.98	0.59-1.62		
**Primary school attainment**	1.27	0.97-1.65			0.79	0.40-1.54		
**Prior incarceration**	**1.50**	**1.21-1.86**			**2.53**	**1.50-4.26**	**3.07**	**1.33-7.07**
***Co-morbidity***								
**HIV infection**	**4.01**	**3.09-5.21**	**3.5**	**2.09-5.74**	**5.46**	**2.49-11.99**	**3.20**	**1.18-8.70**
**HCV infection**	**2.37**	**1.44-3.90**			3.42	0.80-14.61		
**HBV infection (HBcAb+)**	**1.45**	**1.02-2.05**			**3.25**	**1.23-8.58**		
**Psychiatric disorder**	1.06	0.83-1.36			0.70	0.39-1.26		

Multivariate Cox regression with stepwise forward selection method. Statistically significant predictors are in bold (*p* < 0.05).

^1^Multivariate analysis for men included age at admission, injection drug use, prior incarceration, HIV infection, HCV infection and HBV infection.

^2^Multivariate analysis for women included age at admission, injection drug use, prior incarceration, HIV infection and HBV infection.

Among women, age at admission, antecedent of IDU, HBV and HIV infection at admission, and prior incarceration were associated with mortality in the univariate analyses. In multivariate analysis, only HIV infection at admission and prior incarceration were predictors of death (Table 3).

### Mortality trend and causes of death

**Figure 1:**
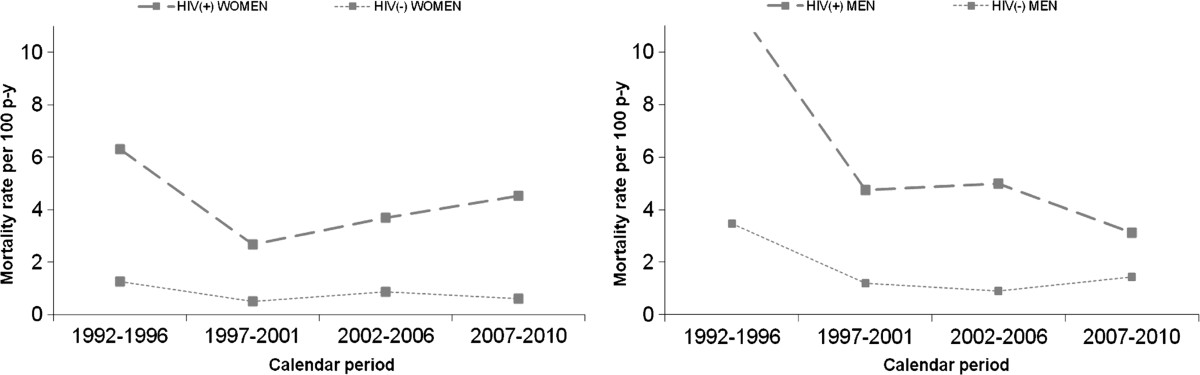
**Sex-specific mortality rates according to four calendar periods and HIV status among patients admitted to a methadone treatment program in metropolitan Barcelona, Spain, 1992–2010.**

Overall mortality rate decreased over time: 7.4 per 100 p-y (95% CI: 6.3-8.8 per 100 p-y) for 1992–1996, 2.7 per 100 p-y (95% CI: 2.2-3.3 per 100 p-y) for 1997–2001, 2.4 per 100 p-y (95% CI: 2.0-2.9 per 100 p-y) for 2002–2006, and 1.9 per 100 p-y (95% CI: 1.6-2.4 per 100 p-y) for 2007–2010. A statistically significant reduction (*p* < 0.005) for all pairwise relative risk of death was observed using the first period as the reference category.Mortality in women slightly increased during recent periods. While the initial mortality rate decreased from 4.4 per 100 p-y (95% CI: 2.6-7.4 per 100 p-y) in 1992–1996 to 1.8 per 100 p-y (95% CI: 0.9-3.2 per 100 p-y) in 1997–2001, it increased to 2.7 per 100 p-y (95% CI: 1.8-3.9 per 100 p-y) in the 2002–2006 period and 2.2 per 100 p-y (95% CI: 1.4-3.5 per 100 p-y) in 2007–2010. This increase in mortality rates was most apparent for the HIV-infected women. Figure 1 shows the temporal trend of mortality rates by sex and HIV status.

To analyze the recent increase in mortality of HIV-infected women, the specific causes of death were reviewed since January 2002. The main causes of death among the 33 HIV-positive women were AIDS-related (53.3%) and liver-related (10%) and few of them (6.7%) died from non-natural causes. The main causes of death among the 116 HIV-positive men who died in the same periods were AIDS-related (33.6%), liver-related (16.8%), and non-natural (18.6%). Overall, there were no statistically significant differences in the causes of death of HIV-positive men and HIV-positive women in the last periods (p = 0.154). However, during the last two periods HIV-positive women showed a higher proportion of AIDS-related deaths than HIV-positive men. (p = 0.048).

## Discussion

This study provides a description of survival in opiate-dependent men and women admitted to MTP and shows that HIV infection is the main predictor of death in this population. Women are more likely to be HIV-positive than men when they are admitted to OST which is consistent with a recent meta-analysis concluding that female IDU are more likely to acquire HIV infection [27]; in the present study IDUs represents 75% of cases.

On the other hand, women from this study were more likely to receive a diagnostic of psychiatric disorder than men which is also consistent with a previous study [22]. Psychiatric comorbidity in this series is high; up to 25% of patients had a mental health disorder before or upon admission to the MTP. However, prevalence of psychiatric disorders in patients with substance abuse is heterogeneous probably because differences in the diagnostic methods or due to the difficulty in obtaining psychiatric diagnoses for drug users [28, 29].

A systematic review of longitudinal studies found that the mortality of heroin-dependent patients varies across countries. Specifically, the pooled crude death rate for all cause mortality was 2.22 × 100 p-y in Western Europe [13]. The mortality of patients in MTP in the present study is even higher, probably due to the impact of HIV/AIDS on survival. Regarding other factors that may influence survival of patients admitted to an OST, we cannot exclude the role that methadone dosage changes and treatment interruptions may have on mortality, as this information that was not available.

In this study, deaths due to non-natural causes, including drug overdose, was significantly higher in men than in women. This finding is consistent with mortality data from European heroin users for whom the male:female ratio for drug overdose deaths is 4:1 [1]. However, a recent study on patients in a MTP demonstrated that the excess mortality due to non-natural causes is higher in women when the standardized mortality ratio is considered [30].

As it has been alredy mentioned, HIV/AIDS was a strong predictor of death, irrespective of gender. These results are consistent with others that have also shown a significant impact of HIV/AIDS on the survival of patients in a MTPs [7, 31–33]. Furthermore, HIV infection in patients on OST is a surrogate of injection drug use; in fact, non-IDUs have a fivefold lower risk of HIV infection than IDUs, as shown in previous studies [34, 35]. In addition, prior incarceration was a risk factor of death in women herein. Imprisonment before being admitted to an OST program may be an indicator of low socioeconomic status and of the severity of drug dependence [36].

This study shows that overall mortality decreased over the two decades analyzed. Significant reductions in the mortality of heroin-dependent patients has been reported in Spain since 1995 [37]; the lower mortality was attributed to the progressive introduction of harm reduction interventions and the dissemination of OST programs.

Another finding of this study is the decline in the number of first time admissions to methadone treatment. Assuming that access to substitution treatment did not change over the study period, there are several potential explanations for the observed decline. First, a lower number of admissions may indicate fewer heroin users in the community; second, the emergence of other heroin substitutes, such as buprenorphine, may reduce the number of patients admitted to the MTP; and third, the irruption of supervised injection facilities in Spain may have produced a shift in attitudes of IDUs, favoring the use of injected heroin in a safe facility. Data from other health indicators support the hypothesis that the decrease in MTP patients may be associated with fewer heroin users in the Barcelona area [38].

This study has several limitations that should be mentioned. First, the number of available clinical and epidemiological variables is limited; thus, it is difficult to determine the impact of additional co-morbidities on survival. However, HIV/AIDS and HCV infections, two diseases with a great impact on survival of patients with a history of IDU, were included as covariates in our analysis [1]. Second, the frequency of poly-drug use while on methadone was not known; it is likely that concurrent use of cocaine and/or alcohol might be associated with an increased risk of death in this population [39]. Third, this study did not analyze neither changes over time regarding methadone dose and treatment interruptions nor changes in HIV and HCV serostatus; in this sense, methadone dose, treatment interruptions and blood-borne infections have been associated with disease outcomes [33, 40].

## Conclusion

In conclusion, this study aimed to analyze disease outcomes in a large cohort of patients admitted to an MTP during 19 years of recruitment and follow-up. The MTP main characteristics have not changed since the program’s inception in 1992 and it is the only MTP in an urban area with a population of 400,000. Adapting treatment delivery to patients’ life conditions and improving the coordination of different health care levels is essential to improve survival of drug-dependent patients treated with methadone.

## Authors’ original submitted files for images

Below are the links to the authors’ original submitted files for images. Authors’ original file for figure 1

## Abbreviations

ART: AntiRetroviral therapy
CI: Confidence interval
HAART: Highly active antiretroviral therapy
HBV: Hepatitis B Virus
HCV: Hepatitis C Virus
HIV: Human immunodeficiency virus
HR: Hazard ratio
ICD: International classification diseases
IDU: Injection drug user
IQR: Inter quartil range
MTP: Methadone treatment program
OST: Opiate substitution therapy
p-y: person-years.
