# Supplementary figures and images for: Sex-specific disease outcomes of HIV-positive and HIV-negative drug users admitted to an opioid substitution therapy program in Spain: a cohort study
Source: BMC Infect Dis. 2014 Sep 17;14:504. doi: 10.1186/1471-2334-14-504 (PMC4261781; doi:10.1186/1471-2334-14-504)

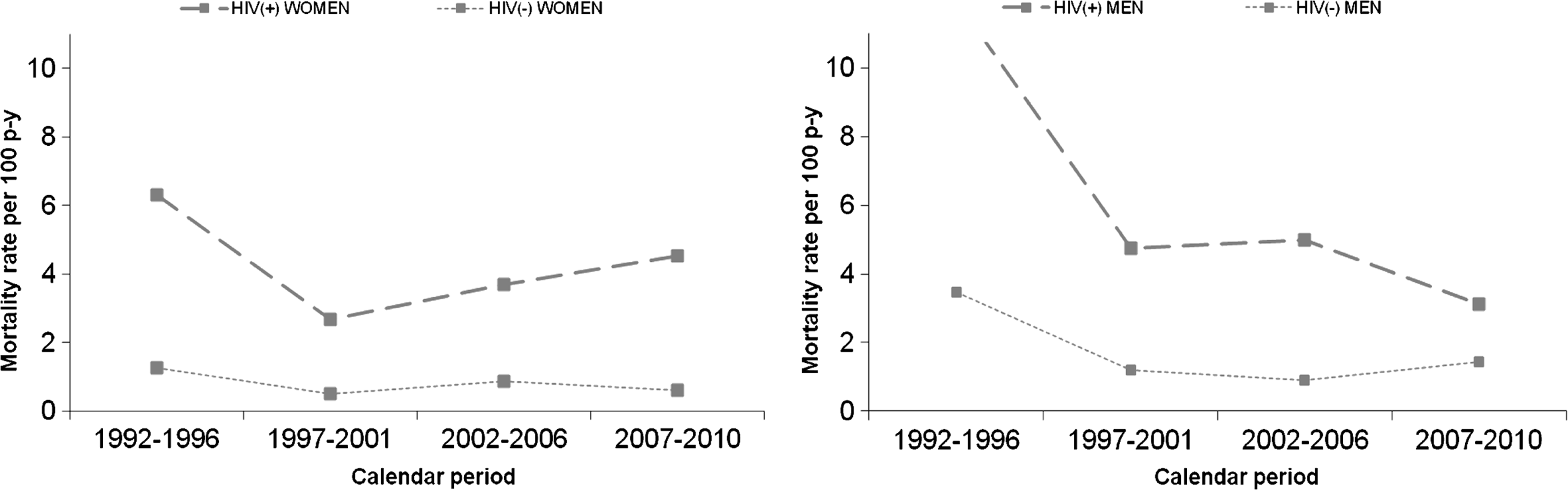

Supplement: Supplementary file 1 — Authors’ original file for figure 1 [file 12879_2014_3835_MOESM1_ESM.tiff]
